# The ELIXIR Human Copy Number Variations Community: building bioinformatics infrastructure for research

**DOI:** 10.12688/f1000research.24887.1

**Published:** 2020-10-13

**Authors:** David Salgado, Irina M. Armean, Michael Baudis, Sergi Beltran, Salvador Capella-Gutierrez, Denise Carvalho-Silva, Victoria Dominguez Del Angel, Joaquin Dopazo, Laura I. Furlong, Bo Gao, Leyla Garcia, Dietlind Gerloff, Ivo Gut, Attila Gyenesei, Nina Habermann, John M. Hancock, Marc Hanauer, Eivind Hovig, Lennart F. Johansson, Thomas Keane, Jan Korbel, Katharina B. Lauer, Steve Laurie, Brane Leskošek, David Lloyd, Tomas Marques-Bonet, Hailiang Mei, Katalin Monostory, Janet Piñero, Krzysztof Poterlowicz, Ana Rath, Pubudu Samarakoon, Ferran Sanz, Gary Saunders, Daoud Sie, Morris A. Swertz, Kirill Tsukanov, Alfonso Valencia, Marko Vidak, Cristina Yenyxe González, Bauke Ylstra, Christophe Béroud

**Affiliations:** 1 Aix Marseille Univ, INSERM, MMG, Marseille, France; 2 European Molecular Biology Laboratory, European Bioinformatics Institute, Wellcome Genome Campus, Hinxton, UK; 3 Department of Molecular Life Sciences and Swiss Institute of Bioinformatics, University of Zurich, Zurich, Switzerland; 4 CNAG-CRG, Centre for Genomic Regulation (CRG), Barcelona Institute of Science and Technology (BIST), Baldiri Reixac 4, Barcelona 08028, Spain; 5 Universitat Pompeu Fabra (UPF), Barcelona, Spain; 6 Barcelona Supercomputing Center (BSC), Barcelona, Spain; 7 Spanish National Bioinformatics Institute (INB)/ELIXIR-ES, Barcelona, Spain; 8 Open Targets, Wellcome Genome Campus, Hinxton, Cambridgeshire, CB10 1SD, UK; 9 Institut Français de Bioinformatique, UMS3601-CNRS, CNRS, Paris, France; 10 Clinical Bioinformatics Area, Fundación Progreso y Salud, CDCA, Hospital Virgen del Rocio, Sevilla, Spain; 11 Research Programme on Biomedical Informatics (GRIB), Hospital del Mar Medical Research Institute (IMIM), Department of Experimental and Health Sciences, Pompeu Fabra University (UPF), Barcelona, Spain; 12 ZB MED Information Centre for Life Sciences, Cologne, Germany; 13 ELIXIR Hub, Hinxton, UK; 14 Luxembourg Centre for Systems Biomedicine, University of Luxembourg, Belvaux, Luxembourg; 15 Szentágothai Research Center, University of Pécs, Pécs, Hungary; 16 Genome Biology, European Molecular Biological Laboratory, Heidelberg, Germany; 17 Orphanet, INSERM, Paris, France; 18 Department of Tumor Biology, Institute for Cancer Research, Oslo University Hospital, Oslo, Norway; 19 Centre for bioinformatics, Department of Informatics, University of Oslo, Oslo, Norway; 20 Department of Genetics, University of Groningen, University Medical Center Groningen, Groningen, The Netherlands; 21 Faculty of Medicine - ELIXIR Slovenia, University of Ljubljana, Ljubljana, Slovenia; 22 Institute of Evolutionary Biology (UPF-CSIC), Catalan Institution for Research and Advanced Studies, Barcelona, Spain; 23 Sequencing Analysis Support Core, Leiden University Medical Center, Leiden, The Netherlands; 24 Institute of Enzymology, Research Centre for Natural Sciences, Budapest, Hungary; 25 Centre for Skin Sciences, University of Bradford, Bradford, UK; 26 Department of Medical Genetics, Oslo University Hospital, Oslo, Norway; 27 Department of Clinical Genetics, Cancer Center Amsterdam, Amsterdam UMC, Vrije Universiteit Amsterdam, Amsterdam, The Netherlands; 28 Catalan Institution of Research and Advanced Studies, Barcelona, Spain; 29 Department of Pathology, Cancer Center Amsterdam, Amsterdam UMC, Vrije Universiteit Amsterdam, Amsterdam, The Netherlands; 30 Département de Génétique Médicale et de Biologie Cellulaire, APHM, Hôpital d’enfants de la Timone, 13385 Marseille, France

**Keywords:** Copy Number Variation, Data analysis, next-generation sequencing, whole genome sequencing, Human Genetics, Oncogenetics, Common Diseases, Federated Human Data

## Abstract

Copy number variations (CNVs) are major causative contributors both in the genesis of genetic diseases and human neoplasias. While “High-Throughput” sequencing technologies are increasingly becoming the primary choice for genomic screening analysis, their ability to efficiently detect CNVs is still heterogeneous and remains to be developed. The aim of this white paper is to provide a guiding framework for the future contributions of ELIXIR’s recently established *h* *uman CNV Community, *with implications beyond human disease diagnostics and population genomics. This white paper is the direct result of a strategy meeting that took place in September 2018 in Hinxton (UK) and involved representatives of 11 ELIXIR Nodes. The meeting led to the definition of priority objectives and tasks, to address a wide range of CNV-related challenges ranging from detection and interpretation to sharing and training. Here, we provide suggestions on how to align these tasks within the ELIXIR Platforms strategy, and on how to frame the activities of this new ELIXIR Community in the international context.

## Introduction

In the late 1950s, Tjio and Levan established that the human karyotype consists of 46 chromosomes ^1^. This was promptly followed by the description of numerical chromosomal abnormalities in Down syndrome and, in less than one year, a new discipline emerged in the field of human genetics: “cytogenetics” ^2^. Shortly after, somatic karyotype alterations were attributed to the identification of “Philadelphia chromosome” in the blood of Chronic Myeloid Leukemia patients ^3^, which were found to be the result of specific underlying chromosomal rearrangements ^4^. From then on, cytogenetics was not only applied to identify heritable chromosomal aberrations, but also established the field of “Cancer Cytogenetics” ^5^, which led to a leap forward in approaching the molecular mechanisms of malignant diseases.

Since these early discoveries, multiple links between chromosomal alterations and diseases have been described. In parallel to those microscopic observations, the identification of the first genomic mutation in humans occurred in 1949 with the discovery of an amino acid change responsible for sickle cell anemia ^6^, while the genetic alteration itself was identified only a few years later by Ingram ^7^. Thanks to the rapidly advancing Sanger sequencing technology ^8^, disease-causing genome variations are now routinely identified and confirmed. However, while both “cytogenetic” and “genomic” alterations in heritable human diseases and cancer are based on alterations of DNA sequence or structure, an epistemological dichotomy remained between the fields of molecular biology and genetics (targeting specific sequence alterations) and cytogenetics (focusing on genetic alterations detected by cytogenetic methods).

In the late 1980s, the development of DNA labeling techniques using the direct or indirect incorporation of fluorescent dyes ^9, 10^ helped to establish the field of “molecular cytogenetics”, in which the hybridization of labeled DNA probes informs about characteristics of chromosomal substrates. Variations of the Fluorescent *In-Situ* Hybridization (FISH) ^11^ technology include interphase FISH (i.e. the hybridization of specific fluorescent probes on interphase nuclei) and reverse *in-situ* hybridization techniques ^8^, in which the labeled DNA sample of interest is hybridized against a substrate consisting of normal metaphase chromosomes. While overall these technologies helped to put “the Genetics back into Cytogenetics” ^12^, the development of Comparative Genomic Hybridization (CGH), a dual-color, whole-genome extension of the reverse *in-situ* hybridization concept was particularly instrumental in the delineation of a type of structural genome variations termed “Copy Number Variations” (CNVs). This category of genome variants encompasses those that range from a few hundred DNA base pairs to such affecting several megabases up to duplications or deletions involving whole chromosomes.

While abundant CNVs have been demonstrated to affect virtually every type of cancer ^13, 14^, they have also been shown to be a large contributor to inherited genome variation and have been shown to impact both genic and intergenic regions alike ^15^.

Since the recognition of CNVs as contributors to the genomic variation landscape, multiple CNVs have been associated with human traits and diseases. Recently, a review by Srebniak *et al*. ^16^ reported, in a meta-analysis of data from 10,314 fetuses, that CNVs were associated with an early-onset syndromic disorder in 0.37% (95% CI, 0.27-0.52%) of cases; with late-onset disease in 0.11% (95% CI, 0.05%-0.21%); and with diseases susceptibility in 0.30% (95% CI, 0.14-0.67%). The prevalence of early-onset syndromic disorders caused by CNVs was thus calculated to be 1:270 ^16^. In parallel, it has been known for years that CNVs account for the majority of disease-causing variation of some genes, as illustrated for example by the *DMD* gene, for which up to 74% and 87% of mutations are deletions or duplications of one or more exons, respectively, for Duchenne and Becker patients ^17^. Additionally to their role in syndromic disorders, CNVs have also been associated with common, polygenic diseases such as obesity ^13, 14^, and mental disorders ^15, 16^.

CNVs are also a major contributor to the somatic mutation landscape in cancer. Specific deletion CNVs may lead to loss of heterozygosity events involving tumor suppressor genes, with a tumor promoting effect demonstrated early on for the somatic loss of wild-type alleles in many inherited cancer syndromes ^18^. In contrast, proto-oncogenes, such as MYCN and ERBB2, are frequently deregulated through chromosomal amplification events, leading to over-expression of the gene products due to a highly increased genomic dosage ^19^. However, while the association between individual duplication and deletion CNVs and tumor-related genes has been determined for many types of cancer, most types of malignancies show recurring CNV patterns with involvement of large genomic regions, beyond a limited number of cancer-associated gene loci.

CNV detection has been performed since the early 1990s using various generations of chromosomal (cCGH) ^20, 21^ and array-based (aCGH) ^22, 23^ CGH technologies, including high-density oligonucleotide arrays and Single Nucleotide Polymorphism (SNP) genotyping arrays ^24, 25^. The current generation of these hybridization technologies is still considered as ‘gold standard’ in order to fulfill the diagnostic needs of clinical cytogenetics laboratories. Their use is highly flexible, as it is easy to design custom arrays, including hundreds of thousands to millions of probes. It is thus possible to use the technology for genome-scale analysis through to region specific analysis.

However, these technologies also have limitations: for example, their inability to distinguish chromosomal abnormalities as tandem, inverted, or translocated duplications; and the probe hybridization process itself which may result in poor sensitivity and precision, depending on probe design and the quality of analyzed DNA. In parallel, it is not currently possible to simultaneously achieve a genome-wide level and a high resolution because of the limited density of the array probes. In addition, as was shown by Haraksingh *et al*., the current generation of arrays still requires careful quantitative comparative analysis for researchers and clinicians to be able to select the appropriate tool for a given application ^26^.

Today, emerging Next-Generation Sequencing (NGS) technologies, especially deep coverage Whole Genome Sequencing (WGS), is quickly becoming the primary strategy for CNV detection in the study of human disease. Because of the ability of this technology to provide a nucleotide level resolution, it can theoretically solve the limitations observed for array-based technologies, and also provide the exact boundaries and localization for a given CNV.

Nevertheless, due to the demanding data analysis workflows and high costs of deep coverage WGS experiments, many laboratories have implemented either low coverage WGS, or the Whole Exome Sequencing (WES) based approach for CNV detection (which stands between array-based approaches and deep WGS). Thus, when targeting the classes of inherited and somatic genome alterations subsumed as CNV for research and clinical applications, one faces a heterogeneous field encompassing different experimental technologies and related bioinformatics methods for data analysis, without a clear ‘gold standard’ serving all heterogeneous applications. Despite these technical challenges, the CNV field is of primary importance for human disease diagnosis and research, including the field of cancer genetics.

In this context, in February 2018, ELIXIR-FR and ELIXIR-ES initiated the creation of the "ELIXIR human CNV Community" (hCNV Community) as a starting point for consolidating this field at the European level, and beyond. The first hCNV Community meeting “The future of hCNV in ELIXIR” took place in Hinxton (UK), as a general, strategically focused, meeting to discuss, prioritize, and map the future of hCNV related activities in ELIXIR. In this white paper, we first summarize the main conclusions of the meeting, and then explain possible future directions for the incorporation of human CNV activities across ELIXIR.

## Meeting: “The Elixir Human Cnv Community”

The initial face-to-face meeting of the ELIXIR human CNV Community took place on September 28 ^th^, 2018 in Hinxton (UK). Attendance was open to any member from any ELIXIR Node, with the limitation of 25 participants at maximum. Remote participation (via teleconference) was offered to those that wished to attend but could not do so in person. There were 21 attendees at the meeting, representing 11 ELIXIR Nodes: EMBL-EBI, France, Germany, Hungary, Luxembourg, Netherlands, Norway, Slovenia, Spain, Switzerland, United Kingdom, and there were also two representatives from the ELIXIR Hub. The meeting started with a presentation from Gary Saunders (ELIXIR Human Data Communities coordinator) who provided a general overview of the current ELIXIR Communities and activities. This initial talk was followed by a series of presentations where each Node summarized their ongoing activities and expertise related to hCNV research. The remainder of the meeting was devoted to an open discussion on the challenges of CNVs and how to address them through activities and tasks building on ELIXIR partners' experience(s). Each activity was mapped to at least one of the five ELIXIR Platforms (Data, Tools, Interoperability, Compute, and Training). In addition, the potential interaction of the hCNV Community with the other ELIXIR Communities and international initiatives was discussed.

## Outcomes and discussion

### Identification of key activities to address CNV challenges

The field of human CNV research is complex and evolving; activities range from CNV detection to their interpretation as potentially pathogenic to common genomic background variation. To simplify this process analysis, seven objectives have been defined by the ELIXIR hCNV Community:

***Objective #1: optimal CNV detection pipelines***

Multiple publications have reported pipelines to detect CNV using micro-array, WES or WGS data ^27, 28^. Nevertheless, as reported by Zare *et al*. ^29^, who evaluated the performance of various CNV detection tools in cancer, there is a low consensus among the tools in calling CNVs, especially from widely used WES experiments: a moderate sensitivity (50 to 80%); a fair specificity (70 to 94%) and a poor false discovery rate (27 to 60%). Similar results were reported by Yao *et al*. ^30^ who concluded that read-depth based programs are still immature for WES-based CNV detection with a low sensitivity and an uncertain specificity. Comparable experiences were revealed by participants of the ELIXIR hCNV workshop, and it was concluded that, even if micro-array technologies provide overall better CNV detection parameters, the wide adoption of NGS technologies represents a true challenge for the accurate detection of CNV. Based on these observations, the need for an extensive assessment and benchmarking of existing tools was established as one of the working areas of the hCNV Community. The objective will be to release a set of sensitive and reliable pipelines, optimized and validated to detect CNV from various high throughput datasets. These pipelines will be available either through ELIXIR compute nodes and/or as stand-alone solutions. Considering the actual performances of available systems, it is anticipated that we will provide a portfolio of tools, each being useful for a specific situation: CNV type; detection technology; disease context.

To reach this objective, three tasks are proposed:

*Task #1.1* will establish the list of available pipelines/software as well as partners’ local solutions to detect CNV from gene panels, WES, low and deep coverage WGS, array CGH, and SNP arrays.

*Task #1.2* will benchmark these systems using datasets from Objective #2 to select the most sensitive, specific, reliable, and rapid systems for each dataset for germline and somatic CNVs. Note that if no system is efficient enough for some conditions, the hCNV partners will develop new system(s) to address community needs. The CNV field is very dynamic and multiple systems are released monthly. We therefore expect to be able to select a combination of tools that will provide enough efficiency to be used for research purposes and eventually diagnostics.

*Task #1.3* will optimize the selected pipelines from Task #1.2 to increase performance on ELIXIR compute nodes and define optimal parameters and guidelines to help end-users to efficiently and reliably detect CNV in various situations through the ELIXIR training platform.

***Objective #2: definition of reference datasets***

The ambition is to produce reference datasets of fully validated somatic and germline CNVs representing a wide range of sample types and experimental technologies. The aim is to provide reference materials available to the community for the comparison and evaluation of pipelines and/or NGS technologies and/or for quality assurance. This will include both digital raw data and, potentially, biomaterials.

To do so, the hCNV Community will establish reference datasets including various CNV (deletions and duplications) of various sizes ranging from a single exon CNV to large genomic rearrangements. Two subsets will be defined for germline and somatic CNVs. These datasets will contain samples with fully validated CNVs by other approaches such as multiplex ligation-dependent probe amplification and quantitative or semi-quantitative PCR. Four tasks are proposed:

*Task #2.1* will be dedicated to WES reference datasets.

*Task #2.2* will be dedicated to WGS reference datasets.

*Task #2.3* will be dedicated to gene panels reference datasets.

*Task #2.4* will be dedicated to CGH/SNP microarrays reference datasets.

During the strategic workshop in Hinxton, participants discussed the GDPR and its impact on reference human datasets. The participation of lawyers and ethics specialists is therefore needed, and this was proposed to be addressed at a more global level by the ELIXIR Human Data Communities as a whole. In case no human reference dataset could be exchanged at a community level, alternatives using other model organisms have been proposed.

As previously mentioned, the NGS technologies are rapidly evolving and therefore the reference datasets will need to be regularly updated.

In the context of tools metrology, i.e. efficiency evaluation and improvement of detection, the Genome in a Bottle initiative has released a set of reference materials which has already been largely used for Short Nucleotide Variation(s) (SNV) ^28, 29^. In a recent paper, Zook *et al.* ^31^ reported the use of a new reference dataset for germline structural variant detection including CNV ^32, 33^. We will therefore adopt these reference samples and use them with all technologies to produce reference datasets.

***Objective #3: data exchange formats***

International collaborative projects require harmonization and standardization of results in order to ensure efficient data aggregation and comparison. Although various international initiatives, such as the GA4GH Genomic Knowledge Standards and Large Scale Genomics Work Streams, are currently addressing aspects of this issue, no robust and exhaustive standard CNV annotation format has emerged so far. To address this issue, the hCNV community identified two tasks:

*Task #3.1* will establish the list of existing formats to describe CNVs and list common and specific features.

*Task #3.2* will develop recommendations to use a standardized format to report CNVs. If a few alternative formats are frequently used, it will provide bioinformatics resources to convert data into the common data exchange format.

hCNV partners notably discussed the adoption of the VCF format and its current limitations for the CNV field. It was concluded that, although this format is well-known by molecular biologists and could therefore be a starting point, it is less frequently used by cytogeneticists. There is therefore a strong need to improve this format and identify other nomenclatures and widely used formats in other communities. It is being recognized that any development or improvement of standards for CNV annotation should be performed in alignment with existing efforts, notably GA4GH work streams and the ELIXIR Interoperability Platform.

***Objective #4: identification of patients with similar genotypes and phenotypes***

Finding similar cases at the clinical level is a key component of clinical diagnosis and research to identify disease-causing genes and to explain genotype/phenotype correlations and intermediate clinical phenotypes. Although perhaps straightforward in the case of common diseases, this is much more of a challenge for the millions of patients affected by a rare disease, as routinely each case is similar only to a handful of patients across Europe. Within the Discovery Work Stream of GA4GH there are standards, such as Beacon ^34^, to establish a federated data discovery network that is able to connect databases of genomic variations and phenotypic data using a common application programming interface (API). The GA4GH Driver Project “ELIXIR Beacons” provides a schema definition and API, together with a reference implementation to allow data owners to add their resources to the Beacon Network, as simply as possible ( https://beacon-project.io). With the recent addition of CNV representation to the Beacon API and planned ontology-based phenotype queries the Beacon ecosystem represents a prime target for the implementation of (CNV related) genotype-phenotype representation and querying. The hCNV group will work towards enabling its use for the envisioned patient discovery, through the support of extended clinical descriptions including enabling and testing of relevant annotation standards (e.g. HPO, NCIt, additional ontologies). To do so:

*Task #4.1* will select ontologies required to efficiently capture phenotypic description useful for data interpretation for any genetic disease.

*Task #4.2* will provide lists of common data elements that should be provided in various situations such as rare disease, oncology, or common diseases.

Various ELIXIR hCNV partners are already strongly involved in the development of ontologies, such as HPO ^35^ and ORDO ^36^ and in the mapping of various ontologies, medical terminologies, vocabularies and nomenclatures. In addition, national databases described in Objective #6, will not only be FAIRified (see below) but also adopt the recommendations from Objective #4 to ensure cross queries and the identification of similar patients.

***Objective #5: creation of innovative tools***

CNVs often involve large genomic regions encompassing multiple genes. In addition, in recessive diseases, CNVs can alter one allele of a specific locus, whilst the second could be altered by SNV. In many situations, it is therefore difficult to identify the single, or multiple, genes harboring variation that is directly associated with a particular phenotype.

The hCNV Community will develop innovative tools to: annotate CNV; facilitate their interpretation through a combinatorial approach; and help to pinpoint key genes in regions of interest. The following tasks have been identified and will most likely evolve as more data become available and as technologies evolve:

*Task #5.1* will define CNV annotations including: type; genotype; genes and transcripts; expression level; exons; regulatory elements; breakpoints/ fusion fragments for WGS only (allowing detection of tandem duplications vs. inverted duplications and translocations).

*Task #5.2* will develop a specific pipeline to interpret duplications as tandem, inverted or translocation duplications may result in very different phenotypes.

*Task #5.3* will develop specific bioinformatics tools to select candidate genes localized in the CNV region by combining genes' annotations and patients' phenotype.

***Objective #6: FAIRification of hCNV services and datasets***

Various CNV national databases, ELIXIR Core Data Resources, and ELIXIR Deposition Databases are currently being developed by ELIXIR hCNV partners. In order to allow interoperability (including discovery), the FAIR principles ( Findable, Accessible, Interoperable, Reusable) ^37, 38^ will be applied to those systems to demonstrate the feasibility and utility of distributed CNV databases. This will respect databases' ownerships and national regulations' compliance while allowing searching for similar patients across the network. To respect and follow these principles, we will ensure that data are:

(i) Findable:

- The data should contain globally unique, resolvable and persistent identifiers.
- Include machine-readable descriptions to support structured search and filtering.

(ii) Accessible:

- Metadata has to be accessible beyond the lifetime of the digital resource (e.g. using BioSchemas).
- Clearly defined regarding the condition for access and security protocols for sharing and accessing data.

(iii) Interoperable:

- Usage of a specific file format
- Extensible machine interpretable formats for data and metadata (e.g. YAML files, JSON-LD)
- Use vocabularies (ontologies) and link with other robust resources
- Integration with FAIR resources

(iv) Reusable:

- Provide licensing, provenance and description on community-standards.

*Task #6.1* will use the French BANCCO database ( http://bancco.fr) developed at Aix Marseille University and the CIBERER (Spanish network for research in rare diseases) database developed at the Fundación Progreso y Salud of Sevillas prototypes to demonstrate the benefits of using the FAIR data principles for CNV in diagnostic and research contexts.

*Task #6.2* will extend the FAIRification to other non-specific CNV databases such as the European Variation Archive (EVA), RD-CONNECT, arrayMap ^39^ and Database of Genomics Variants Archive (DGVa).

***Objective #7: dissemination***

The global adoption of tools and guidelines is strongly linked to the ability to communicate, produce training materials, and train actors as well as patients and the general public.

*Task #7.1* will set-up Jamborees to gather experts' point of view on the various objectives and related tasks and developments.

*Task #7.2* will set-up regular hackathons to ensure smooth developments and benchmarks by various ELIXIR Nodes.

*Task #7.3* will promote the ELIXIR hCNV Community through participation at international meetings, such as GA4GH Plenary. A contact has already been established with the Human Genome Variation Society (HGVS) (see below).

*Alignment with ELIXIR Platforms*

**Figure 1.**
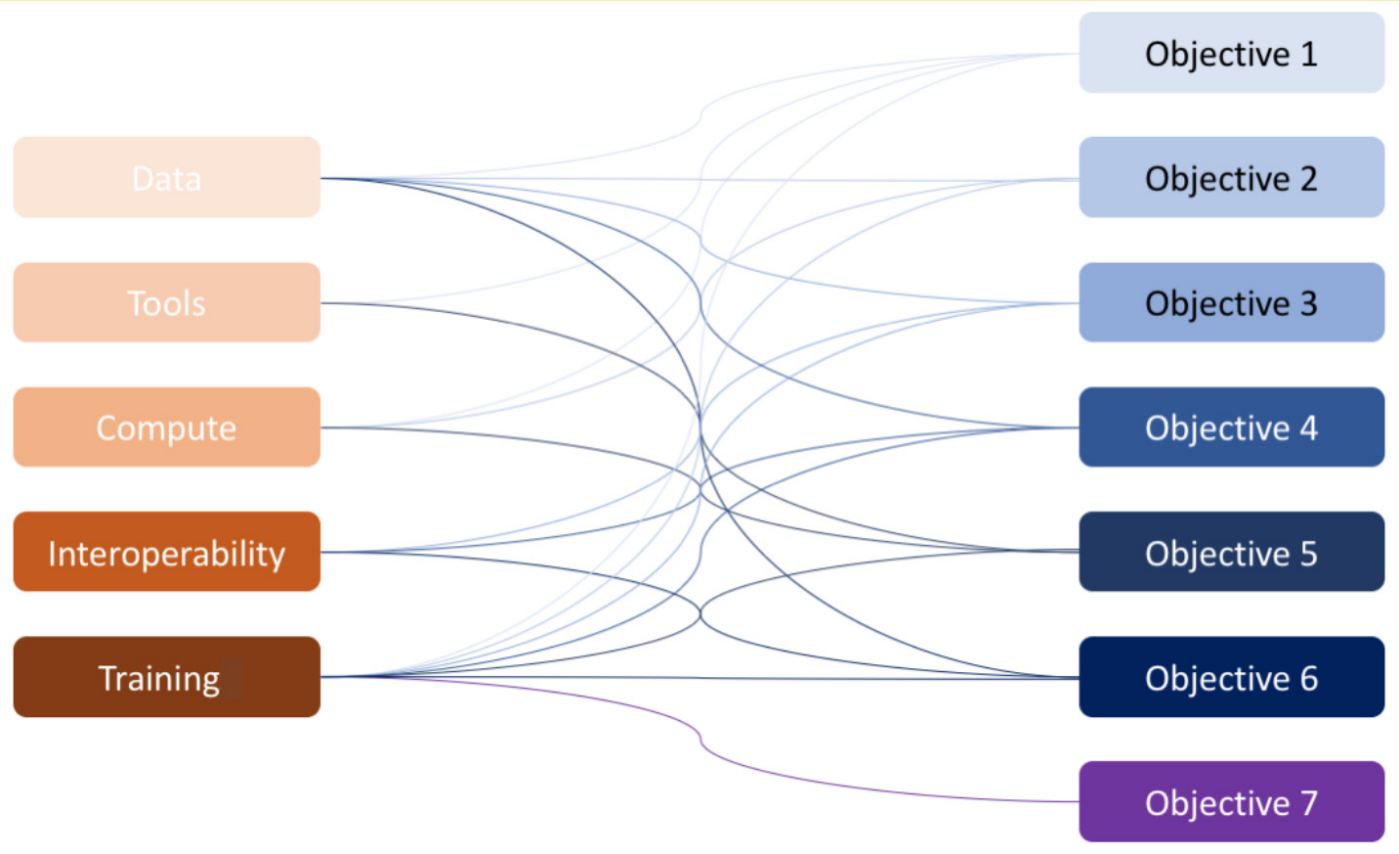
Interactions of the hCNV Community objectives with the ELIXIR platforms. Left: ELIXIR Platforms; Right: ELIXIR hCNV Community objectives.

The ELIXIR hCNV Community’s objectives have many links to the activities of the ELIXIR Platforms ( Figure 1).

Data Platform

The aims of this Platform have been described as to drive the use, re-use, and value of life science data. It aims to do this by providing users with robust, long-term sustainable data resources within a coordinated, scalable and connected data ecosystem. Thus, the ELIXIR hCNV Community that will collect high quality genetic and phenotypic data will establish strong links with the ELIXIR Data Platform by providing Deposition Databases for CNV; and by benefiting from literature data integration and scalable curation provided by the Platform.

Tools and Compute Platforms

As described in Objectives #1 and #5, various reference tools will be validated and/or created by the ELIXIR hCNV Community. Their promotion will be facilitated by inclusion in the "Tools and services registry" bio.tools ( https://bio.tools) ^40^. The activity related to benchmarking will be carried out within the "scientific benchmark and technical monitoring" infrastructure (OpenEbench) ( https://openebench.bsc.es) and the ELIXIR Compute Platform. In addition, tools will be made interoperable and shall be included as Galaxy workflows ^41^ and available to the community through the "Cloud and Computing Resources" from the ELIXIR Compute Platform.

Interoperability Platform

Key to ELIXIR hCNV Community Objective #3 is the development of recommendations to use standardized format(s) to report CNVs. Additionally, Objective #4 will work to extend and harmonize the use of ontologies across the ELIXIR hCNV Community. Both of these objectives align with the “Interoperability with a Purpose at Source” task of the ELIXIR Interoperability Platform Programme for 2019-23.

More generally, in Objective #6, interoperability will be a major component of the hCNV Community. CNV FAIR databases will therefore strongly interact with the ELIXIR Interoperability Platform at multiple levels.

Training Platform

Finally, the global recognition of the ELIXIR hCNV Community's achievements will only be made possible through training, capacity building, and dissemination. A strong link has already been established with this ELIXIR Platform for training coordination and additional collaborations including capacity building will be established as systems, databases and tools are released.

*Alignment with other ELIXIR Communities*

**Figure 2.**
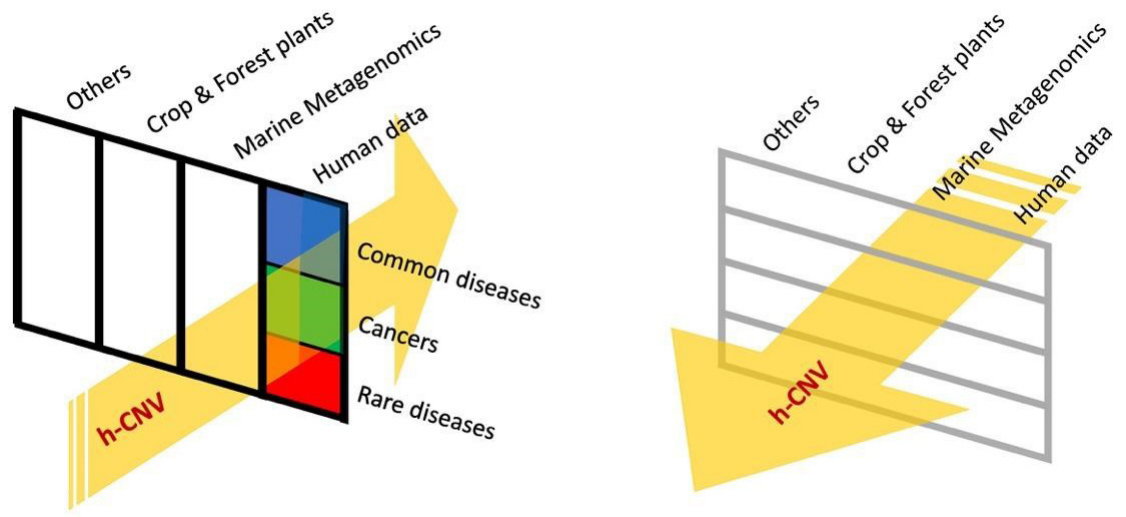
Interactions of the hCNV Community with the ELIXIR Communities. Left: vertical interaction with all of the current ELIXIR Human Data Communities (Federated Human Data and Rare Diseases). Right: It is predicted that the ELIXIR hCNV Community will interact with other ELIXIR Communities, such as Marine Metagenomics and Plant Sciences; relationships to other ELIXIR Communities will be investigated and developed over time.

CNVs are genetic mutations found in all organisms. The ELIXIR hCNV Community will naturally strongly interact with the "Federated Human Data" and "Rare Diseases" ELIXIR Communities ( Figure 2). Moreover, the hCNV community will benefit from the "Federated Human Data" Community thanks to its experience in human data secure access (GDPR) and ethical aspects (ELSI).

Going beyond these, interactions could be established with the ELIXIR Marine Metagenomics and Plant Sciences Communities in the longer term ( Figure 2). In fact, the hCNV detection tools might allow the handling of CNV in plants and marine organisms. Its feasibility will be discussed with these Communities to allow optimal interactions.

In addition, the creation of pipelines and tools will link to the ELIXIR’s Galaxy Community through the implementation of tools and training materials in their platform.

*Alignment with the ELIXIR Industry Programme*

Due to the known involvement in human disease, the topic hCNV is of considerable interest to the scientific industry, for example in the field of personalized medicine. It is therefore of critical importance to ensure that the services we provide are of sufficient quality and fit for purpose for adoption in industry as well as in academia. To facilitate knowledge exchange, we will continue to encourage the participation of industrial partners in the ELIXIR hCNV Community in the role of Community Partner(s). This novel form of engagement is anchored in the ELIXIR Scientific Programme 2019–23 and is part of a comprehensive initiative to embed ELIXIR into the wider ecosystem. Furthermore, we propose to use the ELIXIR Industry Staff Exchange program to allow members of the ELIXIR hCNV Community to work with industry partners on projects of mutual interest in the form of short-term staff exchanges.

*Integration at a global level*

As already described, the use of ontologies, nomenclatures and data exchange formats should be viewed in a more global context. In fact, this dimension has already been addressed by the ELIXIR hCNV Community's partners who have identified international projects and organizations with which relationships will be established. Thus, the GA4GH organization will interact and benefit from the hCNV community at various levels. The International Rare Disease Research Consortium (IRDiRC) will not only benefit from the Community but will also be able to promote its achievements through its members and the "IRDiRC Recognized Resources" labeling program. HGVS has also been approached to participate to their scientific meetings to disseminate the community's achievements.

## Conclusions

We believe that this white paper demonstrates the global need for ELIXIR to establish the human Copy Number Variation Community (hCNV) as it responds to a major challenge of NGS data interpretation in the era of whole genome sequencing both for research and in clinical settings. To do so, we have identified seven objectives ranging from CNV detection to data interpretation and sharing, for which various tasks have been described. The interactions with ELIXIR Platforms and Communities, and worldwide integration in the complex landscape of societies, initiatives and projects, has been addressed to avoid duplication of efforts and ensure fruitful collaborations.

Finally, the growing interest for CNV detection and interpretation in human diseases will ensure the global recognition and expansion of the community and will trigger many interactions both for other ELIXIR Communities (such as Marine Metagenomics and Plant Sciences), and industry.

## Data availability

No data is associated with this article.
