# Supplementary material for: The ELIXIR Human Copy Number Variations Community: building bioinformatics infrastructure for research
Source: F1000Res. 2020 Oct 13;9:ELIXIR-1229. [Version 1] doi: 10.12688/f1000research.24887.1 (PMC8311797; doi:10.12688/f1000research.24887.1)
Supplement: Peer review history

# Peer review: Reviewer response for version 1

**DOI:** 10.5256/f1000research.27459.r88724

**Published:** 2021-07-20

**Authors:** Andrew Lonie

**Affiliations:** 1 University of Melbourne, Melbourne, Vic, Australia

Overall this is an excellent proposal and I only have minor grammatical suggestions. The science case is strong and is described well; the approach is well thought through and well articulated. This would be a very worthwhile ELIXIR community.

**Minor suggestions:**

*Meeting: “The Elixir Human Cnv Community”*

 CNV should be properly capitalised here, as should 'ELIXIR'. And in the previous paragraph, "The Elixir Human Cnv Community” is already introduced and acronymed as 'hCNV Community'.

 Throughout the document, sometimes 'ELIXIR hCNV Community' is used, sometimes 'hCNV Community;, sometimes hCNV. Would be best to standardise.

*Objective #1: *It would be good to discuss how pipelines will be made available for the entire community. It's a complex problem, in practice.

*Objective #2: *It would be good to discuss how the reference datasets will be made available in a FAIR way to the global community.

*Objective #3: *" *Task #3.2* will develop recommendations to use a standardized format to report CNVs. If a few alternative formats are frequently used, it will provide bioinformatics resources to convert data into the common data exchange format." - this sentence doesn't make sense.

*Objective #5: *"The hCNV Community will develop innovative tools to: annotate CNV; facilitate their interpretation through a combinatorial approach; and help to pinpoint key genes in regions of interest. The following tasks have been identified and will most likely evolve as more data become available and as technologies evolve:" 

It should be,

 "The hCNV Community will develop innovative tools to annotate CNVs, facilitate their interpretation through a combinatorial approach, and help to pinpoint key genes in regions of interest.

 The following tasks have been identified and will most likely evolve as more data become available and as technologies evolve:"

*"Task #5.1* will define CNV annotations including: type; genotype; genes and transcripts; expression level; exons; regulatory elements; breakpoints/ fusion fragments for WGS only" - remove the space after "breakpoints/".

 " *Task #5.3* will develop specific bioinformatics tools to select candidate genes localized in the CNV region by combining genes' annotations and patients' phenotype." - this reads a bit strangely. Candidate genes for what? I assume specific rare diseases?

*Objective #6: *"This will respect databases' ownerships and national regulations' compliance" - there should be no trailing apostrophe after regulations; the compliance does not belong to the regulations.

 Nothing much said about FAIRness of all the other artefacts that will be produced (workflows, standards)...

 " *Alignment with other ELIXIR Communities*

 CNVs are genetic mutations..."

Maybe this is better: 

 " *Alignment with other ELIXIR Communities*

 CNVs are genetic variations..."

*"Conclusions": *Again, would be good to standardise on a particular nomenclature of hCNV / hCNV Community / ELIXIR HCNV Community / human Copy number Variation Community and stick with it from the start.

**In summary, this is a very worthwhile community and my comments are generally minor and by way of expression clarifications.**

Is the topic of the opinion article discussed accurately in the context of the current literature?

Yes

Are arguments sufficiently supported by evidence from the published literature?

Yes

Are all factual statements correct and adequately supported by citations?

Yes

Are the conclusions drawn balanced and justified on the basis of the presented arguments?

Yes

Reviewer Expertise:

Bioinformatics, bioinformatics infrastructure, genomics, genetics

I confirm that I have read this submission and believe that I have an appropriate level of expertise to confirm that it is of an acceptable scientific standard.

# Peer review: Reviewer response for version 1

**DOI:** 10.5256/f1000research.27459.r74635

**Published:** 2021-03-22

**Authors:** Antonio Rausell

**Affiliations:** 1 Université de Paris, INSERM UMR1163, Imagine Institute, Clinical Bioinformatics Laboratory, Paris, F-75006, France; 2 Molecular genetics service, AP-HP, Necker Hospital for Sick Children, F-75015, Paris, France

In the white paper "The ELIXIR Human Copy Number Variations Community: building bioinformatics infrastructure for research", Salgado and co-authors present the working plan of the ELIXIR human CNV community. The manuscript summarises the strategy adopted in order to address current challenges in CNV detection and clinical interpretation. First, the introduction reviews the clinical relevance of structural variants in the human genome as well as the evolution of the technologies used for their identification. Second, a total of 7 main objectives are described, which are further detailed into specific tasks. Here, authors propose the development of enhanced CNV detection protocols, reference datasets, computational methods and analyses pipelines, standards for CNV reporting and data exchange and dissemination actions. The manuscript is clearly written and provides a complete and fair overview of the state-of-the-art in the field and of the challenges ahead. Thus, it not only represents a sound and ambitious action plan but, importantly, an insightful reference for current research in human genetics. I congratulate the authors for such effort. I provide below minor comments and suggestions:

1. For non-familiar readers, I would suggest to add a short paragraph introducing ELIXIR and their missions, and to place the human CNV community in such a broader context.
2. The manuscript would benefit from a table summarising the database and software resources previously developed or adopted by the hCNV community that served as a reference or starting baseline for the proposed actions.
3. I missed some mention to recent long-read sequencing technologies. A discussion about the benefits that they may bring to the human CNV field and whether the hCNV community would consider future actions in this direction would be welcome.
4. Also, additional discussion would be welcome as for how the hCNV community effort may indeed boost machine-learning developments oriented to the clinical assessment of CNVs detected from short-read sequencing data. In this direction, the establishment of federated learning strategies across Elixir nodes and associated partners would represent a big step forward. While the manuscript mentions the interaction with the "Federated Human Data" and "Rare Diseases" ELIXIR Communities, the importance of those aspects would deserve more attention. 
5. The use of the term "cytogenetic" as opposed to "genomic" alterations can lead to confusion. I would suggest to rather use structural variants versus single-nucleotide variants and indels.
6. I would suggest rephrasing of the following two sentences: "beyond a limited number of cancer-associated gene loci." [...] "It is thus possible to use the technology for genome-scale analysis through to region specific analysis".

Is the topic of the opinion article discussed accurately in the context of the current literature?

Yes

Are arguments sufficiently supported by evidence from the published literature?

Yes

Are all factual statements correct and adequately supported by citations?

Yes

Are the conclusions drawn balanced and justified on the basis of the presented arguments?

Yes

Reviewer Expertise:

Clinical bioinformatics

I confirm that I have read this submission and believe that I have an appropriate level of expertise to confirm that it is of an acceptable scientific standard.
